# Group Size and Nest Spacing Affect Buggy Creek Virus (Togaviridae: *Alphavirus*) Infection in Nestling House Sparrows

**DOI:** 10.1371/journal.pone.0025521

**Published:** 2011-09-26

**Authors:** Valerie A. O'Brien, Charles R. Brown

**Affiliations:** Department of Biological Sciences, University of Tulsa, Tulsa, Oklahoma, United States of America; University of Georgia, United States of America

## Abstract

The transmission of parasites and pathogens among vertebrates often depends on host population size, host species diversity, and the extent of crowding among potential hosts, but little is known about how these variables apply to most vector-borne pathogens such as the arboviruses (arthropod-borne viruses). Buggy Creek virus (BCRV; Togaviridae: *Alphavirus*) is an RNA arbovirus transmitted by the swallow bug (*Oeciacus vicarius*) to the cliff swallow (*Petrochelidon pyrrhonota*) and the introduced house sparrow (*Passer domesticus*) that has recently invaded swallow nesting colonies. The virus has little impact on cliff swallows, but house sparrows are seriously affected by BCRV. For house sparrows occupying swallow nesting colonies in western Nebraska, USA, the prevalence of BCRV in nestling sparrows increased with sparrow colony size at a site but decreased with the number of cliff swallows present. If one nestling in a nest was infected with the virus, there was a greater likelihood that one or more of its nest-mates would also be infected than nestlings chosen at random. The closer a nest was to another nest containing infected nestlings, the greater the likelihood that some of the nestlings in the focal nest would be BCRV-positive. These results illustrate that BCRV represents a cost of coloniality for a vertebrate host (the house sparrow), perhaps the first such demonstration for an arbovirus, and that virus infection is spatially clustered within nests and within colonies. The decreased incidence of BCRV in sparrows as cliff swallows at a site increased reflects the “dilution effect,” in which virus transmission is reduced when a vector switches to feeding on a less competent vertebrate host.

## Introduction

Two key variables in understanding the transmission of most parasites and pathogens are host population size and the extent of crowding among potential hosts. Numerous studies on directly transmitted parasites (especially ectoparasites) have shown increases in parasite prevalence with increases in vertebrate-host social group size [1]–[8]. Many viral pathogens are known to require minimum population sizes of viable (susceptible) hosts in order to persist in a local area [9]–[12]. As the distance between vertebrate hosts decreases, transmission of macroparasites and some kinds of pathogens increases [8], [10], [13], [14]. However, most of what we understand about the effects of population size and spacing on parasite or pathogen persistence comes from work on directly transmitted ectoparasites or viruses. Little is known about how vertebrate-host group size and spacing affects transmission of vector-borne pathogens [15], [16]; in some of these, transmission may even be reduced in areas of high host density [7], [17].

Most of the arthropod-borne viruses (arboviruses) parasitize multiple vertebrate host species. Their tendency to exploit various hosts makes analysis of the effect of host group size and spacing difficult for most of the vector-borne pathogens. Yet it is important to understand how transmission of arboviruses responds to vertebrate-host social environment: for example, recent work has suggested that prevalence of the medically important West Nile virus (Flaviviridae; *Flavivirus*) may decline in areas that contain high avian host diversity [18], [19]. This may reflect the “dilution effect,” in which numerical increases in less competent amplifying hosts reduce virus transmission because many of the vectors feeding on these hosts fail to become infectious [20]–[22].

In other cases, increases in vertebrate host density and diversity may enhance arbovirus transmission either because (*i*) an abundant host enables vectors to persist even though that host itself may not be a competent amplifier of the virus [23]–[25]; (*ii*) crowding simply increases exposure to horizontally transmitted vectors and for that reason enhances the likelihood of pathogen transmission within a spatial cluster of vertebrate hosts [15], [16], [26]–[28]; or (*iii*) the abundant hosts are more effective virus amplifiers and consequently more vectors may be infected locally [29]–[32]. Few data exist to evaluate these possibilities in most vector-host systems [22]. Information on how vector-borne pathogens such as arboviruses respond to vertebrate-host group size and spacing will also allow us to determine whether these pathogens can represent a cost of sociality in the same way as ectoparasites and directly transmitted microparasites; to date, this is largely unknown. If fitness of more social hosts is reduced by arboviruses, this adds to the suite of parasite-related costs that may constrain vertebrate social evolution in some situations [3], [8], [33], [34].

In this study we take advantage of a relatively simple vector-borne virus/avian host system to explore how group size and spacing of different hosts potentially affects the likelihood of virus infection. We examine evidence for the dilution effect when two different vertebrate host species are present, and use the results to determine whether this arbovirus potentially represents a cost of coloniality for the hosts depending on which species of host is present. Our work is on Buggy Creek virus (BCRV; Togaviridae, *Alphavirus*), an arbovirus in the western equine encephalomyelitis virus complex [35]–[37]. BCRV is transmitted by a swallow bug (Hemiptera: Cimicidae: *Oeciacus vicarius*) to its principal avian hosts, the cliff swallow (*Petrochelidon pyrrhonota*) and the introduced house sparrow (*Passer domesticus*). This virus appears to have little effect on cliff swallows [32], but nestling house sparrows are competent amplifying hosts and exhibit severe pathology related to virus infection [38]. Our analyses here focus on house sparrows and how sparrow colony size and nest spacing potentially affect BCRV prevalence in nestling sparrows, although we also analyze how the presence of cliff swallows may influence the likelihood of virus infection in sparrows. Specifically, we ask whether BCRV prevalence at a bird colony site varies with the number of house sparrows and/or cliff swallows present and for sparrows, how spatial proximity of a nest with infected nestlings affects the likelihood of other nests also having birds positive for BCRV.

## Methods

### Ethics Statement

All handling of animals and procedures done were approved by the Institutional Animal Care and Use Committee of the University of Tulsa, under approval number TU-0036.

### Study Organisms

House sparrows were introduced repeatedly into North America beginning in the 1850s [39] and are now widely distributed and found mainly in peridomestic settings. Sparrows are semi-colonial, often forming aggregations of 2 to 20 nests in close proximity. They are sedentary, remaining at or near breeding sites year-round [40]. House sparrows are multi-brooded, with nesting in our study area beginning in late April and ending in late July, with peak egg laying periods in mid May, late June, and late July. New broods are started soon after earlier ones fail or fledge, and numbers of breeding pairs at a site decline as the summer progresses. Mean (± SE) clutch size for sparrows at latitudes similar to our study area is 4.6–4.8 (±0.8) eggs, and nestlings fledge at 14-17 days of age [40].

Cliff swallows are highly colonial, migratory passerines that breed across much of western North America [41]. They build gourd-shaped mud nests on the sides of cliff faces, inside highway and railroad culverts, and underneath bridges. Nests can be closely spaced, often in direct contact with a contiguous nest. Colonies may contain up to 6000 active nests. The mud nests persist from year-to-year and are frequently repaired and reused by cliff swallows for multiple seasons [42]. Swallows arrive in our study area in early to mid May and typically raise a single brood, with most nestlings fledging by mid July. Individual colonies are highly synchronous and are quickly vacated by swallows after the nestlings fledge. Nestlings are in the nest for about 26 days before fledging [41].

Occupation of cliff swallow nests by house sparrows was thought to be the major reason why cliff swallow populations markedly declined in the eastern United States in the early 20^th^ century [43]. House sparrows likely began to use cliff swallow colonies in our study area after the construction of the interstate highway system in the late 1960's, which provided substrates (bridges, culverts) for cliff swallows to form colonies near humans and thus brought cliff swallows into close contact with house sparrows. Sparrows evict cliff swallows from their mud nests or occupy abandoned nests in colonies where cliff swallows are either present or absent. They fill the nests with grass, feathers, and other materials (making it easy to distinguish a house sparrow nest from a cliff swallow nest in the field) and will breed in them until the nests fall from the substrate.

The swallow bug is a hematophagous nest-based ectoparasite primarily of the cliff swallow. Density of bugs in cliff swallow colonies can be quite high, with as many as 2600 bugs per cliff swallow nest [42]. Swallow bugs are long-lived and can survive without a blood meal for up to three years [44], [45]. The bugs also parasitize house sparrows nesting in cliff swallow nests, and in this way transmit BCRV to them. Bugs feed on birds mostly at night.

Buggy Creek virus is a single-stranded, positive-sense RNA alphavirus [46]. Fort Morgan virus [47], also found in swallow bugs, is a strain of BCRV [37], [48], as probably is a newly described swallow bug virus called Stone Lakes virus [49]. BCRV is ecologically distinct from most alphaviruses in that its vector is the swallow bug, rather than a mosquito [46], [50]–[52]. The virus occurs in two separate lineages [37], [48] that show distinct ecological differences [53]. Prevalence of BCRV in swallow bugs averages ∼25% of bug pools over the whole study area and across different years [15], [54], [55].

### Study Area

Our study area is a 60 × 200 km area largely contiguous with the North and South Platte rivers in western Nebraska, USA, and is centered at the Cedar Point Biological Station (41°13′N, 101°39′W), in Keith County. It also includes portions of Lincoln, Garden, Duel, and Morrill counties. Each year we monitor approximately 170 cliff swallow colony sites, which are occupied to varying degrees by only cliff swallows, cliff swallows and house sparrows together, or only house sparrows. The study area is described in detail by Brown and Brown [42]. We studied house sparrows at colonies in concrete culverts beneath highways or railroads and on the sides of bridges.

### Field Sampling

In May–July 2007, we systematically blood-sampled nestling house sparrows from 21 colony sites throughout the study area. These colonies were chosen both because they contained sparrows and because they were situated in highway culverts where nests could be relatively easily accessed. House sparrow nests were examined for the presence of eggs using a dental mirror and flashlight to see inside the nests. Nests containing eggs were numbered and visited every 2–4 days to determine hatching date and nestling age. We sampled all nestlings in a nest when feasible. Nestlings were between 4–17 days of age when sampled for virus, with all birds bled either once or twice during the nesting period by jugular venipuncture with a 29 gauge insulin syringe. Upon collection, 0.1 mL of blood was placed in 0.4 mL of BA-1 virus diluent [55]. Sampled nestlings were banded with U.S. Geological survey bands and returned to the nest. Blood samples were stored on wet ice in the field, returned to the laboratory, clarified by centrifugation, supernatant removed, and stored at −70°C until screened for virus.

We collected swallow bugs for virus testing by brushing bugs off the exterior of cliff swallow nests into a wide-mouthed collecting jar. Bugs were put into plastic bags and sorted into pools of 100 while alive and stored at −70°C until processed [52], [55]. Because bugs cannot be found in large numbers on the outside of house sparrow nests [32], we collected bugs for this study only from cliff swallow nests.

For each colony site where we blood-sampled house sparrows, we noted whether cliff swallows were present or absent, and recorded cliff swallow colony size (number of active cliff swallow nests) using methods described earlier [42]. House sparrow colony size was defined as the maximum number of simultaneously active nests at any time within the season. At the end of the house sparrow breeding season, we measured the distance (m) between active house sparrow nests and between active house sparrow and active cliff swallow nests in all colonies that were sampled more than once during the season. Distances between colonies were measured using a GPS handheld unit (Garmin International, Inc., Olathe, Kansas). In analyses, the distance to the nearest BCRV-positive house sparrow nest was calculated using the nearest sampled nest that had contained a BCRV-positive house sparrow nestling concurrent with or prior to virus sampling of the focal nest.

### Laboratory Analyses

Viral RNA was extracted from bird sera by first adding 25 µL of thawed sera in BA-1 diluent to 100 µL of a guanidine thiocyanate-based lysis buffer [56]. Bug pools were processed as described in Brown et al. [52]. After the addition of 400 µL of 100% ethanol to the sera or bug-pool homogenate, RNA was extracted using the QIAmp Viral RNA Mini Kit (Qiagen, Valencia, California, USA), following the manufacturer's protocol. A positive BCRV control (derived from swallow bugs) was included in each extraction, and negative controls were placed between every 5 samples. RT-PCR was performed on samples using the OneStep RT-PCR Kit (Qiagen), following the manufacturer's protocol. We used BCRV-specific primers that yielded a 208-bp fragment from the E2 region of the viral genome, as described in Moore et al. [55]. Electrophoresis of product (6.5 µL) on a 4% Nusieve/agarose gel was used to identify any positive samples, using at least one BCRV positive control on each gel and a 100-bp ladder. See Moore et al. [55] for additional details on the RT-PCR methods.

Samples that were initially BCRV-positive by RT-PCR were subjected to plaque assay on Vero cells, as described in Huyvaert et al. [57]. Samples that did not confirm by exhibiting plaque formation on Vero cells were subjected to re-extraction and RT-PCR to confirm presence of viral RNA in the sample [55]. A house sparrow blood sample or bug pool was considered BCRV-positive if either it was RT-PCR-positive on initial screening and confirmed by plaque assay, or it was RT-PCR-positive on initial screening, negative by plaque assay, and positive by RT-PCR on second screening [32], [53]. Some birds were sampled on multiple days during the nestling period: in analyzing prevalence, a bird that tested positive upon first sampling was considered positive for the rest of its nestling period (because presumably an infected bird if surviving cannot become re-infected over such a short period), whereas individuals that were initially negative were also used in calculating prevalence when sampled subsequently (because a negative status can change with time).

### Statistical Analyses

#### Analyses by colony

We used the percentage of nests that were BCRV-positive (defined as ≥ 1 BCRV-positive nestling in the nest at any time) in a colony as the measure of infection at the site over the course of the summer. For these analyses, all nests at each colony site were collapsed into a single data point describing colony-wide prevalence.

#### Analyses by nest

To determine whether nests that contained one BCRV-positive nestling were more likely to contain additional positive nestlings than a nest selected at random, we first calculated the overall percentage of nestlings that were positive (22.2%) from all nests with brood sizes 2–6 (*n* = 853 nestlings). Using this percentage, we generated the expected number of positive and negative nest-mates in the subset of BCRV-positive nests, assuming that positive nest-mates were distributed among these nests in the same proportion as in the total population. The observed number of positive and negative nest-mates in these nests was compared with the expected number using a chi-squared test.

We constructed a set of *a priori* models with nest as the metric using several ecological factors (Table 1) that may have had an effect on the likelihood of infection (≥ 1 BCRV-infected nestling in a nest). We used logistic regression to determine maximum likelihood estimates for each candidate model (PROC LOGISTIC [58]). For the dependent (response) variable, the outcome could be either 0 (BCRV-negative nest) or 1 (BCRV-positive nest). Due to the range of the spatial data and distance-related outliers, potential predictors that used nearest-neighbor distance as a metric (nearest active cliff swallow nest [NNC], nearest active house sparrow nest [NNH], and nearest active BCRV-positive house sparrow nest [NNHP]) were rank-transformed in SAS prior to logistic regression. To test for multicollinearity in predictor variables, we calculated the variance inflation factor for each continuous predictor in the analysis using SAS (PROC REG with options VIF TOL). Models showing overdispersion (Hosmer-Lemeshow test, χ^2^>df; [59]) were not considered in further analysis. We used Akaike's information criterion corrected for small sample size (AIC_c_) to determine the best fitting of our remaining candidate models. The AIC minimizes loss of information in the data by relating the maximum likelihood to the number of parameters in the model [60]. Weight of evidence for each model was determined by normalizing relative likelihood values generated by AIC_c_ using computed Akaike weights (*w_i_*) for all candidate models. We included only models with an Akaike weight within 10% of the highest weight in our confidence set of models [61]. We used the confidence set of models to compute model-averaged parameter estimates for each predictor variable, using the Akaike weights [60].

**Table 1:** Factors potentially influencing the likelihood of ≥ 1 house sparrow nestling in a nest becoming infected with Buggy Creek virus in sparrows nesting in unused cliff swallow nests in western Nebraska.

Factor	Definition
Age	Nestling age (days) at sampling
Date	Date at sampling
Brood	Brood size at sampling
NNH	Distance from nearest house sparrow nest
NNHP	Distance from nearest BCRV-positive house sparrow nest
NNC	Distance from nearest cliff swallow nest

We interpreted effect size and direction in individual predictors using the values of model-averaged partial regression coefficients (β) and their respective 95% confidence intervals and their log-odds ratios (*e* ^β^). We examined the shape of the predicted probabilities of the continuous variables which showed a likelihood of an effect on the response variable (those where the 95% CI did not include zero) by holding all other parameters constant at their mean and varying the focal parameter using the multiple logistic regression equation [62].

## Results

### Effects of Colony Size and Avian Host Species

**Figure 1:**
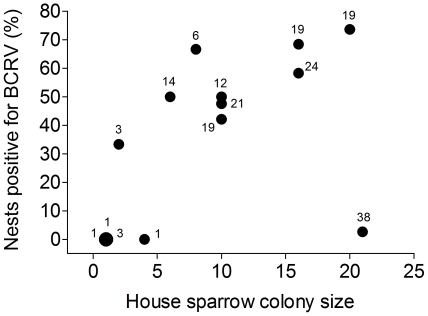
Percentage of house sparrow nests with ≥ 1 nestling positive for BCRV at a site in relation to house sparrow colony size (maximum number of simultaneously active nests). The percentage of positive nests increased with colony size (*n* = 14 colonies; *r_s_* = 0.69, *p* = 0.006). Sample sizes (number of nests) for each colony are shown near the symbols; sample size includes re-nestings and second broods, yielding values higher than the colony size in some cases. The large circle represents three colonies with the same value.

The percentage of house sparrow nests with ≥ 1 nestling infected with BCRV increased with the size of the house sparrow colony at a site (Figure 1). Sparrow colony size was inversely correlated with nest spacing and with average distance to the nearest infected nest within a colony: the mean distance between nests in a colony decreased as colony size increased (*n* = 14 colonies; *r_s_* = −0.89, *p*<0.0001), and the mean distance from a focal nest to the nearest one that contained (or had earlier that season contained) a BCRV-positive nestling decreased as colony size increased (*r_s_* = −0.75, *p* = 0.002). There was an inverse relationship between the percentage of BCRV-positive house sparrow nests in a colony and a nest's mean distance from the nearest house sparrow nest (*r_s_* = −0.79, *p* = 0.0008) and from the nearest nest that contained or had contained a BCRV-positive nestling (*r_s_* = −0.87, *p*<0.0001).

**Figure 2:**
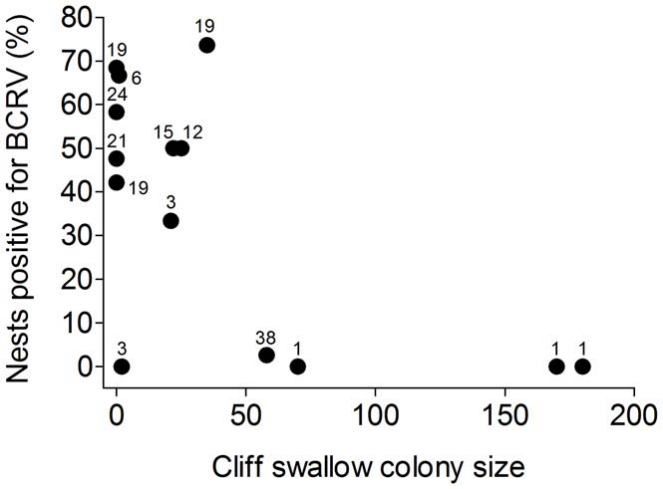
Percentage of house sparrow nests with ≥ 1 nestling positive for BCRV in relation to cliff swallow colony size (maximum number of active nests). The percentage of positive sparrow nests decreased as cliff swallow colony size increased (*n* = 14 colonies; *r_s_* = −0.54, *p* = 0.006). Sample sizes (number of nests) for each colony are shown near the symbols.

Virus prevalence in house sparrow colonies was inversely correlated with cliff swallow colony size (Figure 2). Cliff swallow colony size and house sparrow colony size were not correlated (*n* = 14; *r_s_* = −0.37, *p* = 0.19), so the strong difference between the species (Figure 1 vs. Figure 2) could not simply reflect co-variation between house sparrow and cliff swallow colony size.

**Figure 3:**
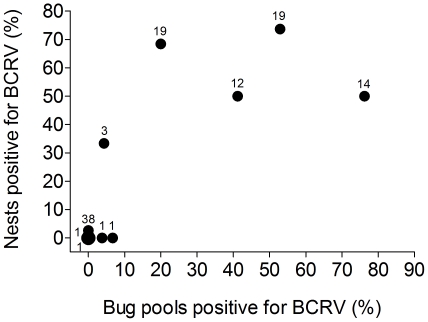
Percentage of house sparrow nests with ≥ 1 nestling positive for BCRV in relation to the percentage of BCRV-positive swallow bug pools in a colony (*r_s_* = 0.77, *p* = 0.009). Swallow bugs were collected at colonies containing both cliff swallows and house sparrows from the outsides of active cliff swallow nests. Sample sizes (number of nests) for each colony are shown near the symbols. The large circle represents two colonies with the same value.

The percentage of BCRV-positive swallow bug pools (collected from bugs on active cliff swallow nests) in a colony containing both cliff swallow and house sparrow nests was directly correlated with the percentage of BCRV-positive house sparrow nests in that colony (Figure 3).

### Effects of Nest Spacing

#### Independence of infection within nests

For nests with brood sizes of 2–6 nestlings containing at least one BCRV-positive nestling (*n* = 68 nests), there were 169 nest-mates of the 68 focal positive nestlings. Assuming an overall infection prevalence of 22.2% of nestlings (see Methods), we should have seen 38 positive and 132 negative nestlings among nest-mates in these nests if infection prevalence was random among nestlings. We observed 69 positive and 100 negative nest-mates, a significant departure from expected (χ^2^_1_ = 13.4, *p*<0.001). Thus, nests with one BCRV-positive nestling were more likely to have positive nest-mates, and less likely to have negative nest-mates, than nests drawn at random. This meant that infection among the nestlings within a nest was not independent, and required that we use the nest (not nestling) as our unit of analysis.

#### Infection prevalence

Analysis of infection prevalence by nest used data from 181 nests where ≥ 1 nestling was tested for BCRV. Tests for multicollinearity on continuous predictor variables revealed minimal overdispersion in the data (VIF<2.0), so no corrections were made. Candidate models without an age, date, and brood-size effect were typically overdispersed (Hosmer and Lemeshow test; χ^2^>df) and ranked well below the models that contained these three parameters. We therefore included age, date, and brood-size variables in all model development. Our confidence set (*n* = 4) of models (Table 2) all included an effect of distance from the nearest BCRV-positive house sparrow nest (NNHP) and an age*date interaction. The highest ranking model showed moderate weight of evidence (*w_i_*  = 0.4693), with the second-ranked model 1.6× less likely to best fit the data. Models without either NNHP or an age*date interaction performed poorly (*w_i_*<10%) and were not included in the confidence set of models (Table 2).

**Table 2:** Model selection results of logistic regression on BCRV infection of nestling house sparrows by nest (*n* = 183 nests).

Model	*k*	AIC_c_	ΔAIC_c_	*w_i_*	Model description
Logit (infected or not) =					
NNC, NNHP, Age*Date	7	202.769	0.000	0.4693	No effect of NNH
Global	8	203.751	0.982	0.2872	Full model
NNHP, Age*Date	5	205.078	2.309	0.1479	No effect of NNH or NNC
NNH, NNHP, Age*Date	7	206.281	3.512	0.0811	No effect of NNC
NNC, NNH, NNHP	7	209.722	6.953	0.0145	No age*date interaction
NNH, NNC, Age*Date	7	246.796	44.027	0.0000	No effect of NNHP
Null	1	252.826	50.057	0.0000	Intercept-only

All models except the intercept-only (null) model included an age, date, and brood effect. Global model included all predictor variables and age*date interaction. Predictor variables are defined in Table 1.

**Figure 4:**
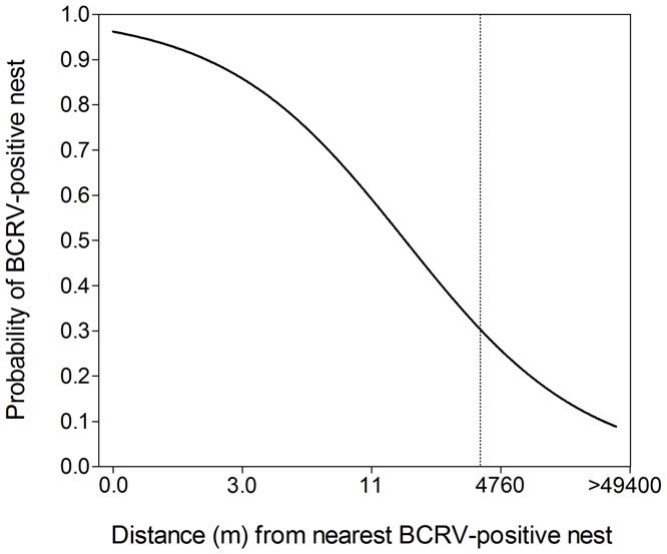
Predicted probability of a house sparrow nest containing ≥ 1 BCRV-positive nestling with distance from the nearest nest containing a BCRV-positive nestling (NNHP) for sparrows nesting at cliff swallow colony sites. Dotted vertical line represents the break between within-colony distances and between-colony distances.

Examination of model-averaged parameter estimates derived from the confidence set of models contained two parameters with likely effects on the response variable when partialed, as indicated by a 95% CI that did not include zero (Table 3). The odds of a house sparrow nest becoming BCRV-positive decreased by 3% with each unit increase in distance from a nest which had contained a BCRV-positive nestling (NNHP; *e* ^β^ = 0.972). The shape of predicted probabilities with an effect of NNHP showed a steep decline in likelihood of becoming infected with only moderate distance from an infected nest (Figure 4), with the probability of infection below 30% for nests in colonies where the closest infected nest was in another colony (Figure 4). The age*date interaction was likely due to a clustering of young nestlings sampled during periods that coincided with highest house sparrow nesting activity in the study area. The effect of the interaction on the outcome variable was low (β = 0.0095, ± SE 0.0035).

**Table 3:** Model-averaged parameter estimates (β), unconditional standard errors (SE), and 95% CI for predictors of BCRV infection by nest from logistic regression analysis (parameters defined in Table 1).

			95% CI	
Parameter	*β*	SE	Upper	Lower
Intercept	5.5261	1.5761	8.5995	2.4526
Age	0.0175	0.1886	0.3853	-0.3502
Date	-0.0470	0.0541	0.0584	-0.1525
Brood	-0.1904	0.2220	0.2425	-0.6233
NNH	0.0047	0.0044	0.0132	-0.0038
**NNHP**	**-0.0286**	**0.0052**	**-0.0184**	**-0.0387**
NNC	-0.0082	0.0044	0.0003	-0.0167
Age*Date	**0.0095**	**0.0035**	**0.0164**	**0.0026**

CI for parameters shown in bold do not include zero.

## Discussion

Our analyses indicate that virus infection of nestling house sparrows was strongly affected by colony size, which host species (house sparrow, cliff swallow) were present at a site, and by nest spacing within a colony. Sparrows were more likely to be infected with BCRV in colonies with larger numbers of active sparrow nests but less likely to be infected at sites with large cliff swallow colonies. The closer a nest was to another nest with infected nestlings, the more likely the nest was to also have infected nestlings, and if one nestling in a nest was infected, there was a greater likelihood that its nest-mates were also infected. The results are a rare illustration of spatial clustering in infection by an arbovirus, and also reveal that the increased risk of virus exposure for sparrows in larger colonies likely represents a cost of coloniality for this species.

### Group Size and the Dilution Effect

To our knowledge there are no previous data for arboviruses showing that per-capita incidence of infection in vertebrate hosts increases with host group size. We earlier reported an increase in infection of swallow bug vectors with BCRV in larger cliff swallow colonies [15] but without information on host infection. The effect of sparrow colony size on virus prevalence in nestling sparrows (Figure 1) probably reflects a higher prevalence of BCRV in bugs at sites with more sparrows, likely driven mostly by the competence of nestling sparrows as hosts for this virus [32]. Because nestling sparrows of all ages can amplify BCRV to titers high enough to infect swallow bugs [63], as more sparrows are present at a site, more virus is amplified and more bugs are infected. In addition, as the number of sparrow nests at a site increases, the availability of blood meals for bugs also increases and promotes bug survival even in the absence of cliff swallows [53], [64]. House sparrows are present at cliff swallow colony sites in our study area to varying degrees throughout the year and raise broods from April to August; in contrast, cliff swallows are in residence at any one site for generally no longer than 8-10 weeks during the summer. Sparrows thus potentially provide a longer period of time during which bugs can feed, become infected, and reproduce, leading to more BCRV infection in bugs and higher bug populations at sites with large numbers of sparrows. Consequently, BCRV may be more likely to persist in the vectors at such sites and more likely to be transmitted to nestling sparrows by bugs. Consistent with this, we found a strong positive relationship between BCRV detected in bug pools (collected from cliff swallow nests) at a site and virus prevalence in nestling sparrows from the same site (Figure 3). In this particular vector-borne system, the horizontal transmission of virus to the avian hosts may mirror the horizontal transmission of bugs among those hosts.

The decline in BCRV prevalence in nestling sparrows both (*i*) at sites with cliff swallows, compared to sites with only house sparrows [32], and (*ii*) as the number of active cliff swallow nests at a site increased (Figure 2), illustrates the dilution effect. As a second vertebrate host is added to the system (in this case, into the bird colony sites), overall virus prevalence in sparrows declines. Two factors likely contribute to this phenomenon: (1) virus amplification decreases because cliff swallows are poor amplifying hosts, rarely showing viremia [32], and (2) the bugs switch their feeding from house sparrows to cliff swallows when the latter are available, thereby reducing the frequency of BCRV transmission to sparrows. The dilution effect as originally conceived [20]–[22] describes situations like this one where a less competent vertebrate host reduces virus transmission by virtue of the vector feeding on it instead of a more competent host. Although there are several clear cases of the dilution effect operating with directly transmitted parasites or pathogens [20], [21], [65]–[67], the BCRV example with house sparrows and cliff swallows is one of the few empirical demonstrations of the dilution effect in arboviruses (see Hess and Hayes [20] for an example with malaria).

Even though the increase in BCRV with sparrow colony size, and the decrease with swallow colony size, were statistically strong patterns (Figs. 1, 2), there was between-site variability. Notably, the largest house sparrow colony studied had almost no virus, and this site also had no cliff swallows until mid-way through the summer (14 June). The single house sparrow nestling that was BCRV-positive in this colony was found only after cliff swallows had colonized the site. Other factors may also influence BCRV prevalence at a given site. A strong predictor of virus prevalence in bugs is the extent to which transient cliff swallows pass through a colony site [54] and introduce infected bugs, and therefore the degree to which a colony site is physically isolated from other colonies (reducing the number of transient birds finding it) may affect observed BCRV prevalence. The large sparrow colony that had little virus was relatively isolated from other active cliff swallow colonies (the two closest were 10.4 and 13.2 km away), in contrast to all other study colonies that were within 5 km of one or more sites containing cliff swallows.

Despite this single uninfected colony, it generally appears that large house sparrow colonies (particularly those with fewer cliff swallows present) with high BCRV prevalence are disadvantageous to sparrows, given the severe effects of this virus on nestlings [38], [63]. Sparrows in larger colonies thus should have lower fitness on average than ones nesting in small groups or solitarily, or those in more isolated areas. The deleterious effects of BCRV on nestling house sparrows [38] would seem sufficient to produce a net cost to coloniality in this species, especially given that there are no known social benefits of group-living (e.g., food-finding, avoidance of predators) for house sparrows [39], [40], [68], [69]. Because of frequent annual turnover among the sparrows occupying cliff swallow colony sites, and the high mortality of BCRV-infected nestlings in our study area [63], it is unlikely that the house sparrow population at most sites develops any degree of herd immunity to BCRV that might ameliorate the virus's severe effects on sparrows. Increasing prevalence of BCRV as colony size increases could be one factor constraining the size of house sparrow colonies at cliff swallow sites.

### Clustering of Virus Infection

We found evidence of non-independence in BCRV infection among the nestlings within a nest, as did Scott et al. [70] for the Fort Morgan strain of BCRV. This is perhaps not surprising if infected bugs remain largely within the same nest as long as nestlings are present, taking repeated blood meals (required each time a bug molts into one of the five instar stages; [71]) from the nestlings present. Contagion of infection among nest-mates is also consistent with direct virus transmission between nestlings in a nest, perhaps through contact with feces or saliva [72]. Some experimental evidence indicates that BCRV can be transmitted directly among house sparrows that share the same cage in the laboratory [57], but further work is needed to determine if this actually occurs in the field.

Logistic regression revealed that the best predictor of whether at least one of the nestling sparrows in a nest would be positive for BCRV was the nearness of another house sparrow nest that either currently had an infected nestling or had earlier had one. This indicates a surprisingly high degree of spatial clustering of virus, especially for one with an arthropod vector. The mechanisms that lead to this clustering are unknown, but could include (*i*) infected bugs moving along the nesting substrate between nests that are relatively close together within a colony, and (*ii*) adult house sparrows moving infected bugs attached to their legs, feet, or feathers [42], [73] from one nest to a nearby nest. Sparrows attempt multiple broods per summer, often using the same nest repeatedly and when using another, settling near their previous nest [40; C. Brown, pers. obs.]. Thus, if they introduce infected bugs to other nests, they would likely do so to nests close by. Having marked birds and monitoring which old swallow nests sparrows choose for successive nesting attempts would help resolve this. Bugs are also capable of moving long distances along the substrate, with one paint-marked bug having moved 65 m within a colony over a 3-day period [42]. Bug movement along the substrate is initiated as soon as the nestlings in a nest fledge or the nest fails [C. Brown and V. O'Brien, pers. obs.]. The high mortality suffered by house sparrow nestlings infected with BCRV [38], [63], combined with the lack of independence of infection within nests, could lead to increased nest failure and thus increased bug movement to nests near those that have failed. This may serve to cluster infected bugs among close neighboring nests.

The spatial clustering of virus-positive nests within the colony means that there is extensive heterogeneity in BCRV infection at a given colony site. Although it is unclear what initially generates this spatial variability, i.e., what seeds virus at a site to start with and why at a particular location within the colony, the consequence is that house sparrows have different fitness expectations depending on where they happen to settle in a colony. This leads to considerable variation within a colony in an individual's expected payoff, and underscores that analyses of the costs and benefits of different group sizes based strictly on colony-wide averages can sometimes be misleading [3], [74].

Compared to most arboviruses, BCRV is unusual in that it is transmitted horizontally by a swallow bug vector rather than a mosquito. However, some mosquitoes can be attracted to larger host colonies [75], and thus even viruses transmitted by mosquitoes might have increased prevalence in larger host groups [76]. That mosquito-associated arboviruses could respond like BCRV to host spacing is suggested by our finding spatial clusters of nestling house sparrows infected with West Nile virus at cliff swallow colony sites [72]. The ecology of BCRV in many ways resembles that of the California group bunyaviruses [77] and some of the tick-borne flaviviruses [78] that maintain relatively stable occurrence in time and space. These arboviruses may exhibit the same responses to vertebrate-host group size and density as we documented here for BCRV.

## References

[1] Hoogland JL (1979). Aggression, ectoparasitism, and other possible costs of prairie dog (Sciuridae, *Cynomys* spp.) coloniality.. Behaviour.

[2] Brown CR, Brown MB (1986). Ectoparasitism as a cost of coloniality in cliff swallows (*Hirundo pyrrhonota*).. Ecology.

[3] Brown CR, Brown MB, Nolan V, Thompson CF (2001). Avian coloniality: progress and problems.. Current ornithology, vol. 16.

[4] Shields WM, Crook JR (1987). Barn swallow coloniality: a net cost for group breeding in the Adirondacks?. Ecology.

[5] Poulin R (1991). Group-living and infestation by ectoparasites in passerines.. Condor.

[6] Poulin R (1991). Group-living and the richness of the parasite fauna in Canadian freshwater fishes.. Oecologia.

[7] Cote IM, Poulin R (1995). Parasitism and group size in social animals: a meta-analysis.. Behav Ecol.

[8] Altizer S, Nunn CL, Thrall PH, Gittleman JL, Antonovics J (2003). Social organization and parasite risk in mammals: integrating theory and empirical studies.. Ann Rev Ecol Syst.

[9] Dietz K (1988). Density-dependence in parasite transmission dynamics.. Parasitol Today.

[10] Anderson RM, May RM (1991). Infectious diseases of humans: dynamics and control..

[11] De Jong MCM, Diekmann O, Heesterbeck H, Mollison D (1995). How does transmission of infection depend on population size?. Epidemic models, their structure and relation to data.

[12] Keeling MJ, Grenfell BT (1997). Disease extinction and community size: modeling the persistence of measles.. Science.

[13] Edmunds WJ, O'Callaghan CJ, Nokes DJ (1997). Who mixes with whom? A method to determine the contact patterns of adults that may lead to the spread of airborne infections.. Proc R Soc Lond B.

[14] Godfrey SS, Bull CM, James R, Murray K (2009). Network structure and parasite transmission in a group living lizard, the gidgee skink, *Egernia stokesii*.. Behav Ecol Sociobiol.

[15] Brown CR, Komar N, Quick SB, Sethi RA, Panella NA (2001). Arbovirus infection increases with group size.. Proc R Soc Lond B.

[16] Nunn CL, Heymann EW (2005). Malaria infection and host behavior: a comparative study of Neotropical primates.. Behav Ecol Sociobiol.

[17] Robert V, MacIntyre K, Keating J, Trape JF, Duchemin JB (2003). Malaria transmission in urban sub-Saharan Africa.. Am J Trop Med Hyg.

[18] Ezenwa VO, Godsey MS, King RJ, Guptill SC (2006). Avian diversity and West Nile virus: testing associations between biodiversity and infectious disease risk.. Proc R Soc Lond B.

[19] Swaddle JP, Calos SE (2008). Increased avian diversity is associated with lower incidence of human West Nile virus infection: observation of the dilution effect.. PLoS One.

[20] Hess AD, Hayes RO (1970). Relative potentials of domestic animals for zooprophylaxis against mosquito vectors of encephalitis.. Am J Trop Med Hyg.

[21] Schmidt KA, Ostfeld RS (2001). Biodiversity and the dilution effect in disease ecology.. Ecology.

[22] Keesing F, Holt RD, Ostfeld RS (2006). Effects of species diversity on disease risk.. Ecol Lett.

[23] Hudson PJ, Norman R, Laurenson MK, Newborn D, Gaunt M (1995). Persistence and transmission of tick-borne viruses: *Ixodes ricinus* and louping-ill virus in red grouse populations.. Parasitology.

[24] Cecere MC, Gürtler RE, Chuit R, Cohen JE (1997). Effects of chickens on the prevalence of infestation and population density of *Triatoma infestans* in rural houses of north-west Argentina.. Med Vet Entomol.

[25] Hudson P, Greenman J (1998). Competition mediated by parasites: biological and theoretical progress.. Trends Ecol Evol.

[26] Komar N (1997). Reservoir capacity of communally roosting birds for eastern equine encephalitis (EEE) virus..

[27] Hodgson JC, Spielman A, Komar N, Krahforst CF, Wallace GT (2001). Interrupted blood-feeding by *Culiseta melanura* (Diptera: Culicidae) on European Starlings.. J Med Entomol.

[28] Kent R, Juliusson L, Weissmann M, Evans S, Komar N (2009). Seasonal blood-feeding behavior of *Culex tarsalis* (Diptera: Culicidae) in Weld County, Colorado, 2007.. J Med Entomol.

[29] Kilpatrick AM, Daszak PD, Jones MJ, Marra PP, Kramer LD (2006). Host heterogeneity dominates West Nile virus transmission.. Proc R Soc Lond B.

[30] Kilpatrick AM, Kramer LD, Jones MJ, Marra PP, Daszak P (2006). West Nile virus epidemics in North America are driven by shifts in mosquito feeding behavior.. PLoS Biol.

[31] Kelly DW, Paterson RA, Townsend CR, Poulin R, Tompkins DM (2009). Parasite spillback: a neglected concept in invasion ecology?. Ecology.

[32] O'Brien VA, Moore AT, Young GR, Komar N, Reisen WK (2011). An enzootic vector-borne virus is amplified at epizootic levels by an invasive avian host.. Proc R Soc B.

[33] Møller AP, Allander K, Dufva R, Blondel J, Gosler A, Lebreton JD, McCleery R (1990). Fitness effects of parasites on passerine birds: a review.. Population biology of passerine birds.

[34] Schmid-Hempel P (1998). Parasites in social insects..

[35] Weaver SC, Kang W, Shirako Y, Rümenapf T, Strauss EG (1997). Recombinational history and molecular evolution of western equine encephalomyelitis complex alphaviruses.. J Virol.

[36] Powers AM, Brault AC, Shirako Y, Strauss EG, Kang W (2001). Evolutionary relationships and systematics of the alphaviruses.. J Virol.

[37] Pfeffer M, Foster JE, Edwards EA, Brown MB, Komar N (2006). Phylogenetic analysis of Buggy Creek virus: evidence for multiple clades in the western Great Plains, United States of America.. Appl Environ Microbiol.

[38] O'Brien VA, Meteyer CU, Ip HS, Long RR, Brown CR (2010). Pathology and virus detection in tissues of nestling house sparrows naturally infected with Buggy Creek virus (Togaviridae).. J Wildl Dis.

[39] Lowther PE, Cink CL, Poole A (2006). House Sparrow (*Passer domesticus*).. The birds of North America.

[40] Anderson TR (2006). Biology of the ubiquitous house sparrow..

[41] Brown CR, Brown MB, Poole A, Gill F (1995). Cliff swallow (*Hirundo pyrrhonota*).. The birds of North America.

[42] Brown CR, Brown MB (1996). Coloniality in the cliff swallow: the effect of group size on social behavior..

[43] Stoner D (1939). Parasitism of the English sparrow on the northern cliff swallow.. Wilson Bull.

[44] Smith GC, Eads RB (1978). Field observations on the cliff swallow, *Petrochelidon pyrrhonota* (Vieillot), and the swallow bug, *Oeciacus vicarius* Horvath.. J Wash Acad Sci.

[45] Rannala BH (1995). Demography and genetic structure in island populations..

[46] Hopla CE, Francy DB, Calisher CH, Lazuick JS (1993). Relationship of cliff swallows, ectoparasites, and an alphavirus in west-central Oklahoma.. J Med Entomol.

[47] Calisher CH, Monath TP, Muth DJ, Lazuick JS, Trent DW (1980). Characterization of Fort Morgan virus, an alphavirus of the western equine encephalitis virus complex in an unusual ecosystem.. Am J Trop Med Hyg.

[48] Padhi A, Moore AT, Brown MB, Foster JE, Pfeffer M (2008). Phylogeographical structure and evolutionary history of two Buggy Creek virus lineages in the western Great Plains of North America.. J Gen Virol.

[49] Brault AC, Armijos MV, Wheeler S, Wright S, Fang Y (2009). Stone Lakes virus (family Togaviridae, genus *Alphavirus*), a variant of Fort Morgan virus isolated from swallow bugs (Hemiptera: Cimicidae) west of the Continental Divide.. J Med Entomol.

[50] Rush WA, Francy DB, Bailey RE (1981). Seasonal changes in susceptibility of a population of swallow bugs (Hemiptera: Cimicidae) to Fort Morgan virus.. J Med Entomol.

[51] Rush WA, Francy DB, Smith GC, Cropp CB (1980). Transmission of an arbovirus by a member of the family Cimicidae.. Ann Entomol Soc Am.

[52] Brown CR, Brown MB, Padhi A, Foster JE, Moore AT (2008). Host and vector movement affects genetic diversity and spatial structure of Buggy Creek virus (Togaviridae).. Mol Ecol.

[53] Brown CR, Padhi A, Moore AT, Brown MB, Foster JE (2009). Ecological divergence of two sympatric lineages of Buggy Creek virus, an arbovirus associated with birds.. Ecology.

[54] Brown CR, Brown MB, Moore AT, Komar N (2007). Bird movement predicts Buggy Creek virus infection in insect vectors.. Vector-Borne Zoo Dis.

[55] Moore AT, Edwards EA, Brown MB, Komar N, Brown CR (2007). Ecological correlates of Buggy Creek virus infection in *Oeciacus vicarius*, southwestern Nebraska, 2004.. J Med Entomol.

[56] O'Brien VA, Moore AT, Huyvaert KP, Brown CR (2008). No evidence for spring re-introduction of an arbovirus by cliff swallows.. Wilson J Ornithol.

[57] Huyvaert KP, Moore AT, Panella NA, Edwards EA, Brown MB (2008). Experimental inoculation of house sparrows (*Passer domesticus*) with Buggy Creek virus.. J Wildl Dis.

[58] SAS Institute (2004). SAS/STAT 9.1 user's guide, version 8.2..

[59] Hosmer DW, Lemeshow S (2000). Applied logistic regression..

[60] Burnham KP, Anderson DR (2002). Model selection and multimodel inference: a practical information-theoretic approach.. 2nd ed.

[61] Royall RM (1997). Statistical evidence: a likelihood paradigm..

[62] Cohen J, Cohen P, West SG, Aiken LS (2003). Applied multiple regression/correlation analysis for the behavioral sciences.. 2nd ed.

[63] O'Brien VA (2009). Ecological interactions between arboviruses and their avian hosts..

[64] Brown CR, Strickler SA, Moore AT, Knutie SA, Padhi A (2010). Winter ecology of Buggy Creek virus (Togaviridae, *Alphavirus*) in the central Great Plains.. Vector-Borne Zoo Dis.

[65] Telfer S, Bown KJ, Sekules R, Begon M, Hayden T (2005). Disruption of a host-parasite system following the introduction of an exotic host species.. Parasitology.

[66] Suzán G, Marcé E, Giermakowski JT, Mills JN, Ceballos G (2009). Experimental evidence for reduced rodent diversity causing increased hantavirus prevalence.. PLoS One.

[67] Carver S, Kuenzi A, Bagamian KH, Mills JN, Rollin PE (2010). A temporal dilution effect: hantavirus infection in deer mice and the intermittent presence of voles in Montana..

[68] Summers-Smith D (1963). The house sparrow..

[69] McGillivray WB (1980). Nest grouping and productivity in the house sparrow.. Auk.

[70] Scott TW, Bowen GS, Monath TP (1984). A field study of the effects of Fort Morgan virus, an arbovirus transmitted by swallow bugs, on the reproductive success of cliff swallows and symbiotic house sparrows in Morgan County, Colorado, 1976.. Am J Trop Med Hyg.

[71] Usinger RL (1966). Monograph of Cimicidae..

[72] O'Brien VA, Meteyer CU, Reisen WK, Ip HS, Brown CR (2010). Prevalence and pathology of West Nile virus in naturally infected house sparrows, western Nebraska, 2008.. Am J Trop Med Hyg.

[73] Brown CR, Brown MB (2004). Empirical measurement of parasite transmission between groups in a colonial bird.. Ecology.

[74] Kosciuch KL, Langerhans RB (2004). Evolution of coloniality via commodity selection: what about variance?. Auk.

[75] Brown CR, Sethi RA (2002). Mosquito abundance is correlated with cliff swallow (*Petrochelidon pyrrhonota*) colony size.. J Med Entomol.

[76] Kent R, Juliusson L, Weissmann M, Evans S, Komar N (2009). Seasonal blood-feeding behavior of *Culex tarsalis* (Diptera: Culicidae) in Weld County, Colorado, 2007.. J Med Entomol.

[77] Reisen WK (1990). North American mosquito-borne arboviruses: questions of persistence and amplification.. Bull Soc Vector Ecol.

[78] Brown CR, Moore AT, Young GR, Komar N (2010). Persistence of Buggy Creek virus (Togaviridae, *Alphavirus*) for two years in unfed swallow bugs (Hemiptera: Cimicidae: *Oeciacus vicarius*).. J Med Entomol.

